# Supplementary figures and images for: NlpC/P60 peptidoglycan hydrolases of Trichomonas vaginalis have complementary activities that empower the protozoan to control host-protective lactobacilli
Source: PLoS Pathog. 2023 Aug 16;19(8):e1011563. doi: 10.1371/journal.ppat.1011563 (PMC10461829; doi:10.1371/journal.ppat.1011563)

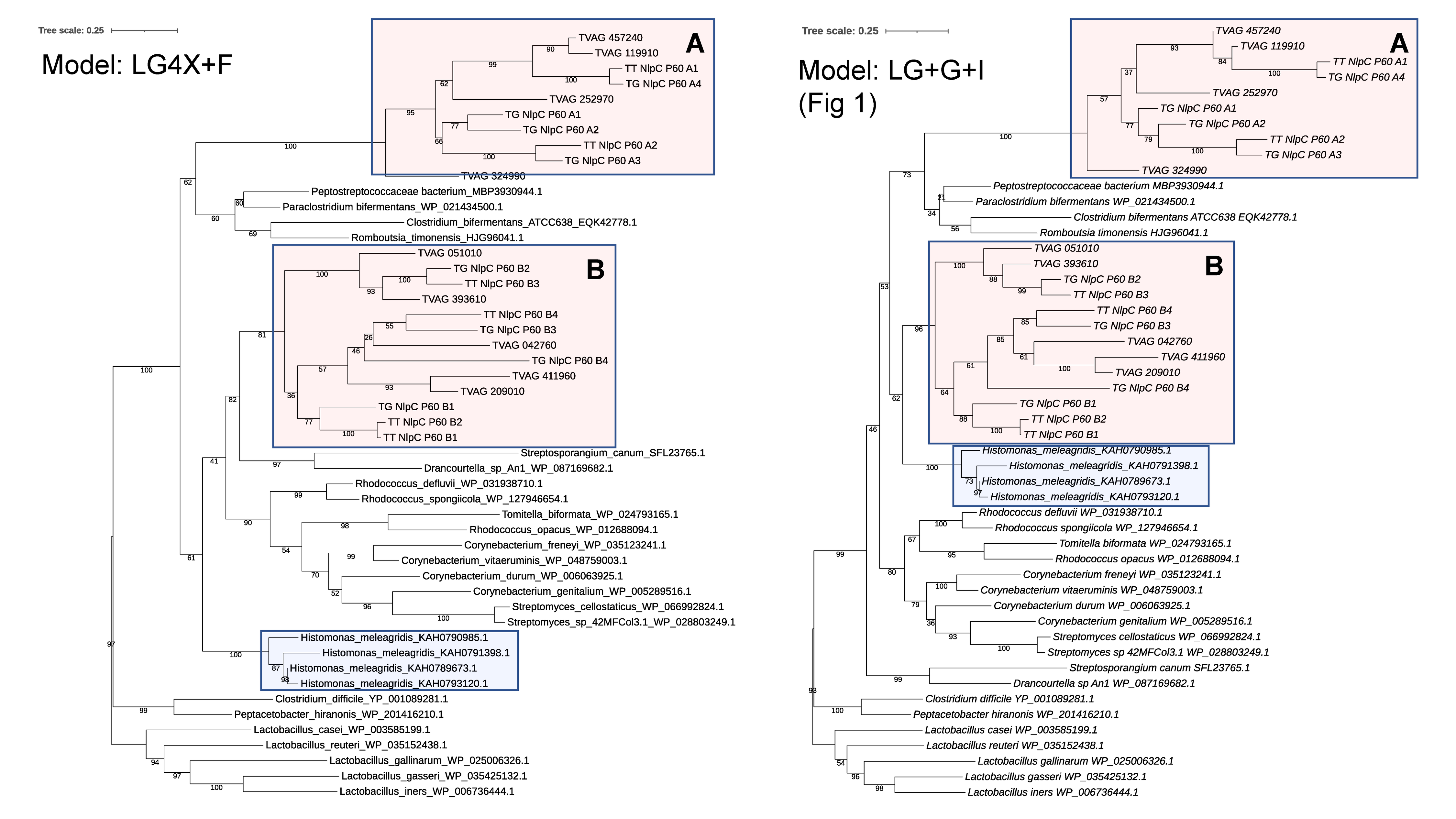

Supplement: S1 Fig — Different models and taxa sampling were used to investigate the phylogenetic relationships between the identified NlpC/P60 protein sequences from the three species of Trichomonas: T. vaginalis, T. tenax and T. gallinae. Sequences from the fourth Trichomonad Histomonas meleagridis were also included in addition to close bacterial homologues to the Trichomonads homologues. Maximum likelihood based phylogenetic inferences used: (i) the best fitting identified homogenous model for the protein alignment using the automatic model selection function as implemented in iq-tree (see Material and Methods sections for mode details) (model LG+G+I, shown on the right and that also corresponds to the phylogeny shown in Fig 1) or (ii) protein mixture models, one such phylogeny is shown on the left (LG4X+F) that allow different amino acid composition and evolutionary rates across the alignment. The protein mixture models were used, as they are considered to be more reliable in extracting phylogenetic signal from divergent sequences. The branches with the lower support values (from 1000 ultrafast bootstraps) are also those corresponding to sections of the phylogenies that are sensitive to the model used. These differences between these two phylogenies highlight the lack of phylogenetic signal in that short alignment of only 103 residues. (TIF) [file ppat.1011563.s001.tif]

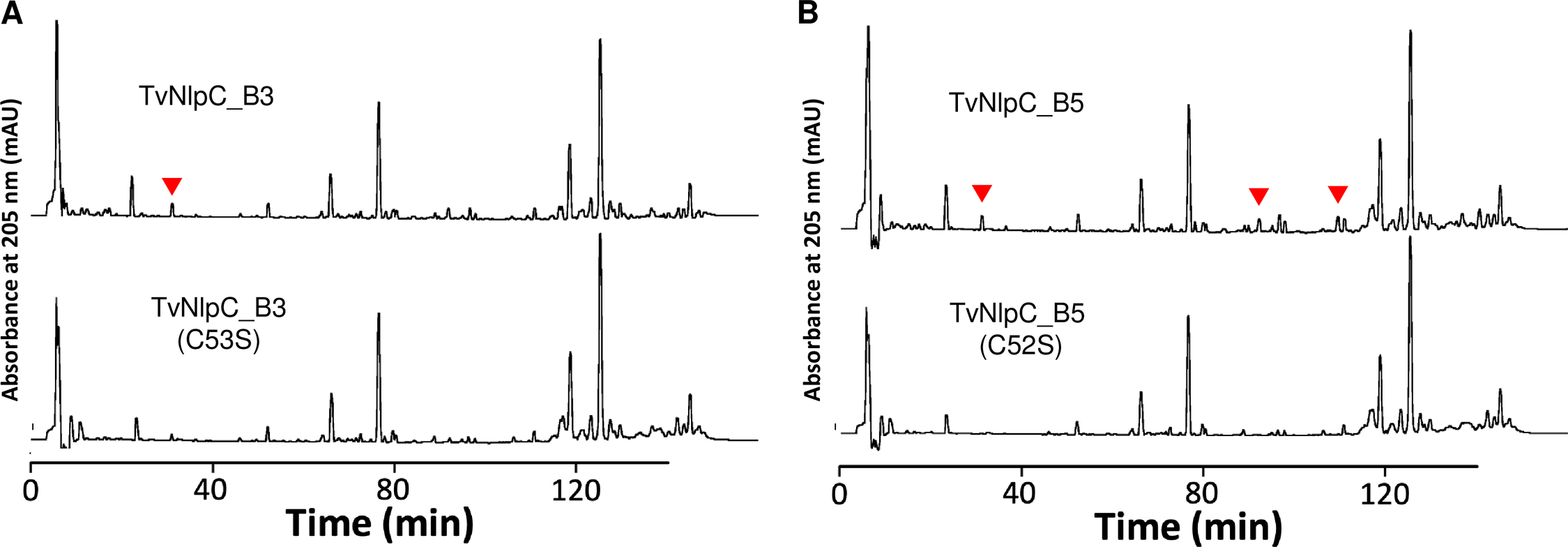

Supplement: S2 Fig — The catalytic-active and -inactive versions of TvNlpC_B3 (A) and TvNlpC_B5 (B), as indicated, were incubated with PG from L. gasseri. Muropeptides were released by cellosyl, reduced with sodium borohydride and separated by HPLC. Cleavage products (red arrows), that are absent from the -inactive enzyme controls, indicate activity of these enzymes against the PG of L. gasseri. (TIF) [file ppat.1011563.s002.tif]

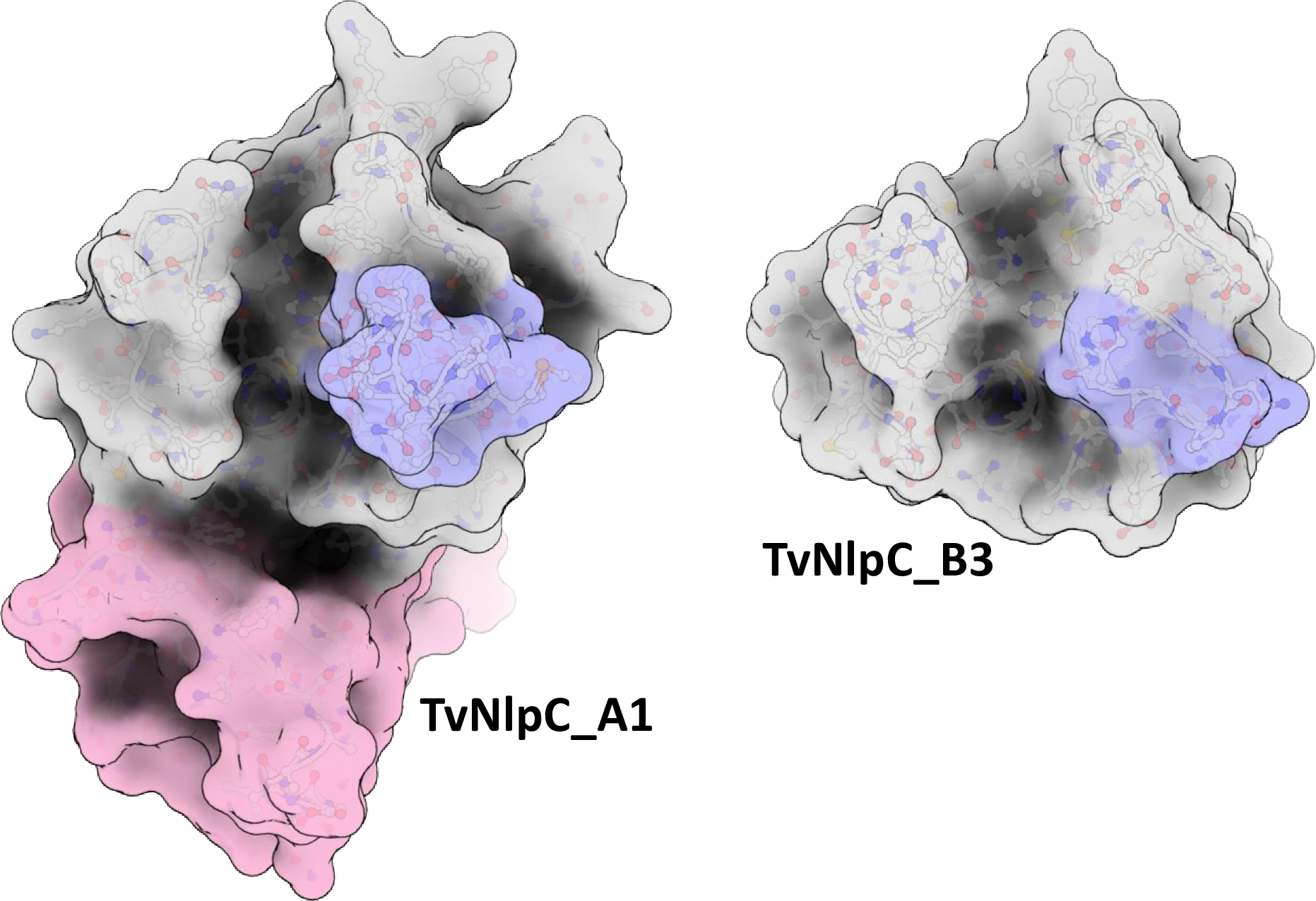

Supplement: S3 Fig — This comparison shows that TvNlpC_B3 exhibits a more open and accessible groove shape than TvNlpC_A1, including an absence of the nearby extended loop structure between strands S1 and S2 (highlighted blue). A nearby SH3 domain (highlighted pink) modulates the groove in TvNlpC_A1. (TIF) [file ppat.1011563.s003.tif]

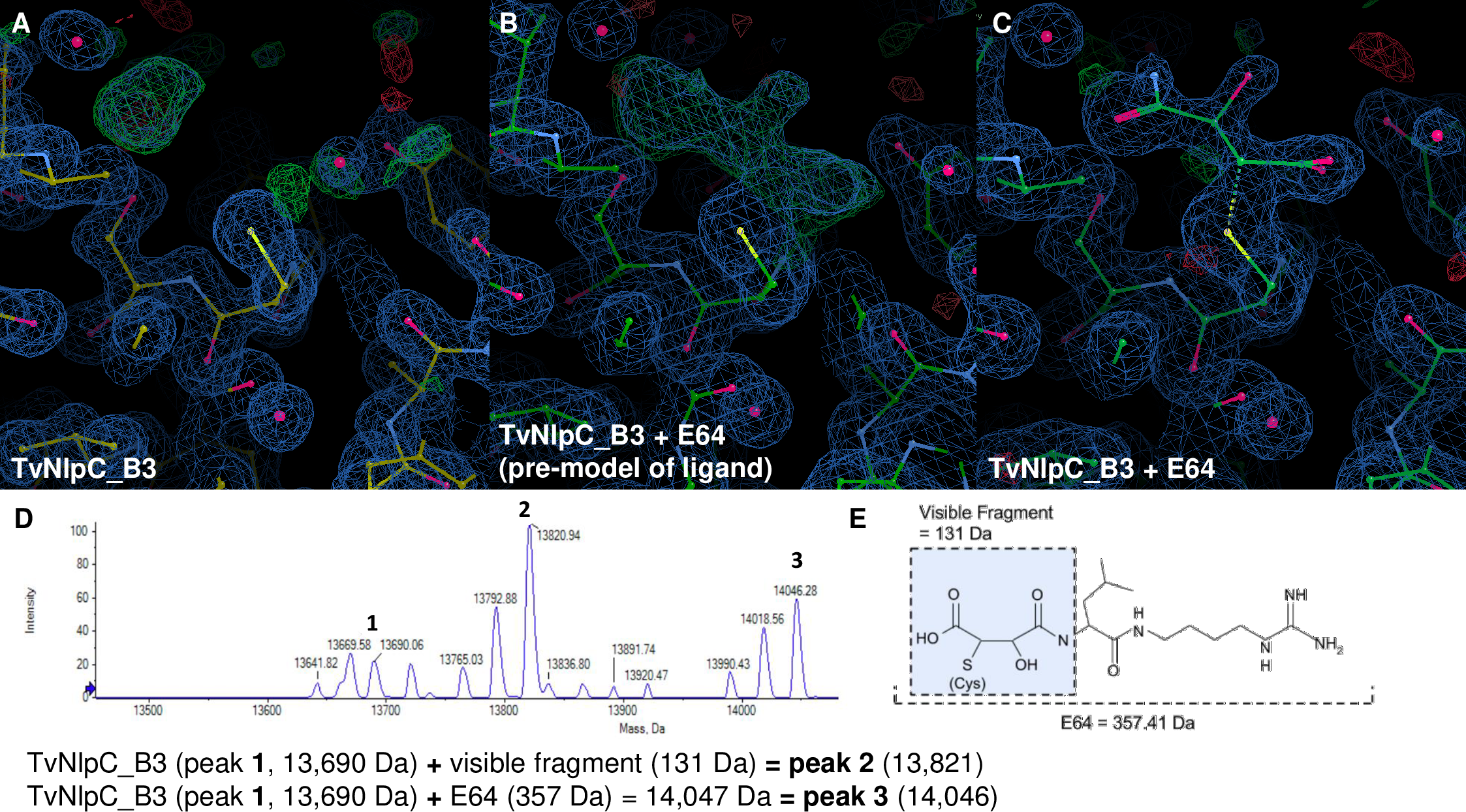

Supplement: S4 Fig — For reference, 2Fo-Fc is shown at 1.0 sigma blue, positive and negative Fo-Fc at 3.0 sigma are shown in green and red respectively. (A) Apo TvNlpC_B3, (B) TvNlpC_B3 with E64 prior to and after building of E64 fragment, and (C) final model of TvNlpC_B3 with E64 fragment modelled. (D) Mass spectroscopy analysis of TvNlpC_B3 incubated with E64. Three major peaks were observed corresponding to TvNlpC_B3 (peak 1), TvNlpC_B3 and the E64 fragment (peak 2) and TvNlpC_B3 and E64 (peak 3). (E) The fragment identified in both the crystallography and mass spectroscopy analysis corresponds to a 131Da fragment of E64. (TIF) [file ppat.1011563.s004.tif]
