# Supplementary material for: NlpC/P60 peptidoglycan hydrolases of Trichomonas vaginalis have complementary activities that empower the protozoan to control host-protective lactobacilli
Source: PLoS Pathog. 2023 Aug 16;19(8):e1011563. doi: 10.1371/journal.ppat.1011563 (PMC10461829; doi:10.1371/journal.ppat.1011563)
Supplement: S2 Table — (DOCX) [file ppat.1011563.s006.docx]

|  | **TvNlpC B3 : 8EV4** | **TvNlpC B3 with E64 : 8EV5** |
| --- | --- | --- |
| **Wavelength (Å)** | 0.9537 | 0.9537 |
| **Resolution range (Å)** | 36.3 - 1.30 (1.32 - 1.30) | 45.2 - 1.65 (1.68 - 1.65) |
| **Space group** | P 4_1_ 2_1_ 2 | P 4_1_ 2_1_ 2 |
| **Unit cell (Å, °)** | 34.0 34.0 181.4  90 90 90 | 34.0 34.0 180.9  90 90 90 |
| **Total reflections** | 347147 (8872) | 281058 (6855) |
| **Unique reflections** | 27691 (1356) | 13779 (640) |
| **Multiplicity** | 12.5 (6.5) | 20.4 (10.7) |
| **Completeness (%)** | 100.0 (99.2) | 99.6 (98.5) |
| **Mean I /σ(I)** | 14.1 (1.5) | 14.1 (1.60) |
| **Wilson B-factor** | 9.5 | 14.3 |
| **R-merge** | 0.099 (1.11) | 0.136 (1.36) |
| **R-meas** | 0.103 (1.20) | 0.139 (1.43) |
| **R-pim** | 0.029 (0.46) | 0.029 (0.43) |
| **CC_1/2_** | 0.998 (0.500) | 0.999 (0.567) |
| **CC*** | 0.999 (0.816) | 0.999 (0.851) |
| **R-work** | 0.158 (0.250) | 0.183 (0.323) |
| **R-free** | 0.170 (0.280) | 0.198 (0.254) |
| **CC(work)** | 0.968 (0.812) | 0.968 (0.758) |
| **CC(free)** | 0.953 (0.823) | 0.996 (0.837) |
| **Number of Atoms** | 934 | 937 |
| **macromolecules** | 868 | 859 |
| **ligands** | 0 | 14 |
| **solvent** | 66 | 64 |
| **Protein residues** | 115 | 115 |
| **RMS(bonds)** | 0.009 | 0.009 |
| **RMS(angles)** | 1.28 | 1.27 |
| **Ramachandran favoured (%)** | 97.35 | 98.23 |
| **Ramachandran allowed (%)** | 2.65 | 1.77 |
| **Ramachandran outliers (%)** | 0.00 | 0.00 |
| **Rotamer outliers (%)** | 0.00 | 0.00 |
| **Clashscore** | 0.58 | 0.58 |
| **Average B-factor** | 14.59 | 19.26 |
| **macromolecules** | 13.78 | 18.32 |
| **ligands** | n/a | 40.20 |
| **solvent** | 25.24 | 27.32 |

Statistics for the highest-resolution shell are shown in parentheses.
