# Supplementary material for: NlpC/P60 peptidoglycan hydrolases of Trichomonas vaginalis have complementary activities that empower the protozoan to control host-protective lactobacilli
Source: PLoS Pathog. 2023 Aug 16;19(8):e1011563. doi: 10.1371/journal.ppat.1011563 (PMC10461829; doi:10.1371/journal.ppat.1011563)
Supplement: S2 Data — (DOCX) [file ppat.1011563.s009.docx]

Active site residues in **Bold underline**,

N-terminal signal sequence truncations in ~~strike-through~~,

Final constructs include N-terminus ‘Gly-Pro-Gly’ motif from remnant HRV-3C cut-site.

**TvNlpC B3**

*GPG*

1 ~~MLSFFFAAAL SARRR~~HERRE VPNASGSAIL NVAKSRIGKQ YMSGGTGPDL FD**C**SGLVLYS 60

61 HNQCGVYGVP RVAKDQARGG KAGSGAAGDV VYFGNPAH**H**V GICCGDGSMV **H**APRPGKTVC 120

121 ILKIAYMKES YGYRRYY 137

## TvNlpC B5

*GPG*

1 ~~MLGLLFTLAC SRRTHH~~HQLA SNGLGDQILA VAQSKKGCPY VYGGNGPNSF D**C**SGLVKYCH 60

61 NKCGINNIAR TASQIAKGGK SGNGSPGDVA YYGNPAY**H**VG ICVNSAGMI**H** APKPGDVVKY 120

121 QAFKYYRPKG FRRYW 135
