# Supplementary material for: NlpC/P60 peptidoglycan hydrolases of Trichomonas vaginalis have complementary activities that empower the protozoan to control host-protective lactobacilli
Source: PLoS Pathog. 2023 Aug 16;19(8):e1011563. doi: 10.1371/journal.ppat.1011563 (PMC10461829; doi:10.1371/journal.ppat.1011563)
Supplement: Attachment — Submitted filename: Response to editor and reviewers.docx [file ppat.1011563.s010.docx]

Dear editor and reviewers.

We are thankful for the effort and contribution on revising our initial manuscript submission. We have done substantial revisions following the generally positive comments, constructive criticisms and recommendations from all of you. We are confident that the revised manuscript has improved substantially.

**EDITOR.** In the light of the reviews, the editor invited us to re-submit a revised manuscript following the reviewer’s recommendation including the specified changes:

**(1)** Showing that **“**NlpC/P60 hydrolases produced by *T. vaginalis* break down the peptidoglycan of Lactobacillus”.

**Answer.** In this revised manuscript, we added the results of the *in vitro* activity of TvNlpC_B3 and TvNlpC_B5 on the PG of *L. gasseri*. This was included in the Results as S2 Fig (lines 200-201), and in Methods (lines 516-517). It is important to note that our *in vitro* assays have a limitation. The native structural features of the bacterial cell wall may be required for the specificity of these enzymes. Therefore, a low or lack of activity *in vitro* against a particular purified PG may not indicate necessarily less or no activity *in vivo*. This was recently illustrated by Lisboa et al. 2021 [53] whereby authors evaluated bacterial specificity of the NlpC/P60 PnpA based on assays with insoluble sacculi. Instead of purified PG, sacculi might preserve some of the native structural features of the cell wall that PnpA is required for recognising the PG with specificity. To this point, we expanded this discussion in the revised manuscript by re-structuring the last two paragraphs of the Discussion (lines 371-383).

**(2)** Comparing “killing effect of *T. vaginalis* on several bacteria, not just lactobacilli”.

**Answer.** In this revised manuscript, we added the results of *T. vaginalis* activity over the vaginal bacterium *Gardnerella vaginalis* as part of Fig 6, which Fig and legend were modified accordingly. The results were described in the last paragraphs of Results (lines 274-292), with two notes in the Abstract (lines 25-26 and 36-37) and one in Discussion (lines 352-355). In the subsection of Methods “Single and mixed cultures of protozoan and lactobacilli”, now re-named to “Single and mixed cultures of protozoan and vaginal bacteria”, changes were done accordingly to accommodate the cultivation of *G. vaginalis*. As described in our original manuscript, *G. vaginalis* is a key species of the human vaginal microbiome in dysbiosis (i.e. to the opposite spectrum of *L. gasseri*), in addition to other vaginal bacterial species of this microbiome. The interactions of *T. vaginalis* with these two bacteria have been examined in the last years by our group and others, with findings converging to this parasite exhibiting antagonistic and synergistic interactions with *G. vaginalis* and *L. gasseri* respectively. For instance, using human ectocervical cells pre-colonized with vaginal bacteria, *T. vaginalis* was shown to reduce the number of lactobacilli but not of *G. vaginalis* or other dysbiotic bacteria [17]. In agreement, the new results within this revised manuscript indicate that this bactericidal activity of *T. vaginalis* is preferentially exerted against the homeostatic *L. gasseri*. It is clear though, and now discussed in lines 362-364 of the revised manuscript, that “Further studies are needed to understand the complementary activities of all nine NlpC/P60 enzymes of *T. vaginalis* and their preference for targeting vaginal bacteria with specificity”.

**(3)** Addressing “whether phagocytosis is responsible for lactobacillus killing”.

**Answer.** This is a valid point. In our original manuscript, and contrary to the surface/secreted TvNlpC_A1 and A2 [21], we report TvNlpC_B3 and _B5 in *T. vaginalis* organellar fraction by Western blots and as punctate spots in *T. vaginalis* cells by immunofluorescence. While it is possible that phagocytosis may be relevant to this interaction, we feel that examining phagocytosis is beyond the scope of this manuscript. However, in the revised manuscript, we now offer to the readers and detail this possibility using appropriate literature [46-49] highlighting that phagocytosis by *T. vaginalis* is underappreciated aspect of its biology (lines 338-350). The involvement of phagocytosis in this microbial interaction is an open avenue for further investigation.

**(4)** Reporting “*T. vaginalis* viability at the endpoint of experiments where the parasite is incubated at high concentrations for long periods”.

**Answer.** We apologize for this typo in our original submission. We meant to say 2-5 x 10^5 cells per ml and not 10^6. Under our experimental conditions, highly viable *T. vaginalis* cultures reach a maximum of 1-2 x 10^6 cells/ml. We have corrected this typo in the revised manuscript (line 425).

**REVIEWER #1.** To our understanding, this reviewer raised minor comments (listed #1 to 5) which were addressed below.

**(1)** The reviewer asked about the possible involvement of phagocytosis in this microbial interaction.

**Answer.** We thought this was a very interesting observation, since NlpC_B3 and NlpC_B5 were detected in the organellar fraction by Western blots and as punctate spots in protozoan cells by immunofluorescence. It is indeed possible that phagocytosis may be relevant to this interaction. We feel, however, that examining phagocytosis is beyond the scope of this manuscript. In the revised manuscript, we now offer and detail to the readers this possibility using appropriate literature [46-49] and highlighting that phagocytosis is underappreciated aspect on the biology of *T. vaginalis* (text expanded from lines 338-350).

**(2)** Quote: “Lane 206-277. This section is very long. Is it possible to reduce the number of words?”

**Answer**. We agree with the reviewer. We have made changes to this section of the manuscript by removing repetition and overly detailed descriptions of the active site groove that the reader can explore for themselves via the PDB models. This has reduced the section by approximately 150 words.

**(3)** Quote: “Lane 383. The authors state that the culture conditions are microaerophilic. They should report at least the height of the tubes, to demonstrate the distance of the cultured cells from the interface of air and oxygen”

**Answer.** We have changed the description in Methods to be specific, quote: “Both microorganisms were grown at 37°C without agitation in microaerophilic condition, i.e. in standard 15 ml Falcon tubes (12 cm high) filled up with 15.5 ml of media to reduce oxygen exchange.” (lines 417-419).

**(4)** Quote “Lane 389: The number of cells is very high: the concentration of 2-5 x 10^6, especially for long incubation periods, is not physiological for *T. vaginalis*. The authors should report cell viability under these conditions”

**Answer.** We apologize for this typo in the original submission. We meant to say 2-5 x 10^5, and we have corrected in this re-submission (line 425). Under our experimental conditions, highly viable *T. vaginalis* cultures reach a maximum of 1-2 x 10^6 cells/ml.

**(5)** Quote “Lane 396. Is the sample collected after shaking the cultures? If the cultures are not shaken, the bacteria tend to sediment.”

**Answer.** This is a good point. We have reported that *L. gasseri* ATCC 9857 self-aggregate and sediment in conical tubes previously [14]. In this study, our experiments were done in multi-well plates but cultures were mixed by pipetting before samples were collected. In the revised manuscript, this have now been clarified along this line (lines 429-430), quote: “Cultures were mixed by pipetting, before collecting samples for plating on agar.”

**REVIEWER #2**. To our understanding, this reviewer raised three major comments (listed #1 to 3) and some minor comments (listed #4-10) which were addressed below.

**Reviewer #2, major comments**

**(1)** The reviewer questioned whether Tv_NlpCs might break down the peptidoglycan of *Lactobacillus*; as we only showed *in vitro* hydrolase activity on peptidoglycan of *E. coli* strains. Quote: “Why not *Lactobacillus* PG? If TvNlpC_B3 and B5 can cleave *Lactobacillus* PG, it will provide insights on mechanism of controlling *Lactobacillus* population.”.

**Answer**. This is a valid point. To address this point, we added the results of the *in vitro* activity of TvNlpC_B3 and TvNlpC_B5 on the PG of *L. gasseri* in the revised manuscript. This was included in the Results as S2 Fig (lines 200-201), and in Methods (lines 516-517). It is important to note that our *in vitro* assays have a limitation. The native structural features of the bacterial cell wall may be required for the specificity of these enzymes. Therefore, a low or lack of activity *in vitro* against a particular purified PG may not indicate necessarily less or no activity *in vivo*. This was recently illustrated by Lisboa et al. 2021 [53] whereby authors evaluated bacterial specificity of the NlpC/P60 PnpA based on assays with insoluble sacculi. Instead of purified PG, sacculi might preserve some of the native structural features of the cell wall that PnpA is required for recognising the PG with specificity. To this point, we expanded this discussion in the revised manuscript by re-structuring the last two paragraphs of the Discussion (lines 371-383).

**(2)** The reviewer questioned whether the bactericidal activity of *T. vaginalis* is observed against vaginal bacterial other than lactobacilli.

**Answer.** This is a great observation. There are many key species in the human vaginal microbiome in eubiosis and dysbiosis. The interactions of *T. vaginalis* with these bacteria have been examined in the last years, with studies by our group and others converging to the findings that this parasite and vaginal bacterial in eubiosis and dysbiosis display antagonistic and synergistic interactions respectively. For instance, using human ectocervical cells pre-colonized with vaginal bacteria, *T. vaginalis* was shown to reduce the number of lactobacilli but not of dysbiotic bacteria [17]. As described in our manuscript, *Gardnerella vaginalis* is a key species of the human vaginal microbiome in dysbiosis (i.e. to the opposite spectrum of *L. gasseri*). For the revision of this manuscript, we have now done co-incubation experiments with protozoan and *G. vaginalis*. The new results within the revised manuscript indicate that this bactericidal activity of *T. vaginalis* is preferentially exerted against the homeostatic *L. gasseri*, which is in agreement with the aforementioned publication. These findings were shown as part of Fig 6, which Fig and legend were modified accordingly. The results were described in the last paragraphs of Results (lines 274-292), with two notes in the Abstract (lines 25-26 and 36-37) and one in Discussion (lines 353-355). In the subsection of Methods “Single and mixed cultures of protozoan and lactobacilli”, now re-named to “Single and mixed cultures of protozoan and vaginal bacteria”, changes were done accordingly to accommodate the cultivation of *G. vaginalis*. We acknowledge in our manuscript that nine enzymes and a few vaginal bacterial species imply open avenues for many studies in the future. Thus, we added in lines 361-363 of the revised manuscript that “Further studies are needed to understand the complementary activities of all nine NlpC/P60 enzymes of *T. vaginalis* and their preference for targeting vaginal bacteria with specificity”.

**(3)** The reviewer suggested complementing the ‘gain-of-function’ phenotypes shown in here with ‘loss-of-function’ experiments.

**Answer.** We appreciate the reviewer’s suggestion. While there is much apparent redundancy and complementarity across this family of NlpC/P60 enzymes in *T. vaginalis*, the lack of a suitable technology for targeting multiple genes in this organism is a huge technical challenge for achieving ‘loss-of-function’ phenotypes across most or all nine genes. Although CRISPR/Cas9 has been applied in *T. vaginalis* recently (Janssen BD et al. Sci Rep. 2018; 8:270), only two drug markers for *T. vaginalis* (i.e. neomycin- and puromycin-resistant genes) are available for drug selection.

**Reviewer #2, minor comments**

**(4)** Quote “Figure 2B. Quantification of cell clumps size does not provide much of additional value as there is no statistical comparison and there is a variability in sizes.”

**Answer.** The reviewer is correct. We have now applied a pairwise test to show that the increase on clump size is statistically significant between 2 and 4 or 2 and 8 hours of incubation (p < 0.01), but not between 4 and 8 hours (p = 0.03). The Fig and legend were changed accordingly.

**(5)** Quote “Figure 3B. Scale bars are missing.”

**Answer.** We appreciate for this point being raised, which was fixed in the resubmission*.*

**(6)** Quote “Line 102: Authors claim that crystal structure was obtained with bound ‘biological ligand’. E64 inhibitor is broad spectrum cysteine peptidase inhibitor (papain, cathepsin B, cathepsin L, calpain and staphopain) isolated from Aspergillus japonicus. E64 is not a natural ligand for TvNlpCs.”

**Answer**. This is a valid point. The term ‘biological’ was removed from three parts of the manuscript (lines 104, 217 and 316). The inhibitor was specifically identified now in the revised Abstract as well for clarification (line 32).

**(7)** Quote “Figure 3. WB. Anti-Fd. More details are required to describe what is Anti-Fd (from material and method section it is ferredoxin).”

**Answer.** We agree. The legend of this figure was modified for clarification: “probed with antibodies against the HA-tagged protein (Anti-HA) or the organellar marker ferredoxin (Anti-Fd).”

**(8)** Quote “Figure 4. Typos for labels: B3 and B3 (C53S) mislabeled as B5 and B(C52S).”

**Answer**. We are glad the reviewer spotted this typo, which was fixed in the resubmission.

**(9)** Quote “Line 233. Typo: ‘S2 Fig’ instead of ’Fig. S2’”

**Answer**. This reads S2 Fig.

**(10)** Quote “Line 249. Though LC-MS data of TvNlpC_B3 with bound E64 inhibitor was mentioned, no data was demonstrated to confirm covalent biding of E64 to TvNlpC_B3. Moreover, a good negative control would be LC-MS data of TvNlpC_B3(C53S) + E64.

**Answer**. This is a good point, and we are thankful for the suggestion. We have updated supplementary S4 Fig to include mass spectrometry data. The updated S4 Fig shows the MS peaks corresponding to TvNlpC_B3 bound to both full length E64 and the E64 fragment. We have also included improved views of electron density showing the fragment covalently bound to the active site Cys53 of TvNlpC_B3. The S4 Fig legend has been expanded to detail these changes.
